# Multilaboratory Comparison of Pneumococcal Multiplex Immunoassays Used in Immunosurveillance of Streptococcus pneumoniae across Europe

**DOI:** 10.1128/mSphere.00455-19

**Published:** 2019-11-27

**Authors:** Bob Meek, Nina Ekström, Bjørn Kantsø, Rachael Almond, Jamie Findlow, Jenna F. Gritzfeld, Charlotte Sværke Jørgensen, Karl Ljungberg, Fredrik Atterfelt, Manou R. Batstra, Kevin Andeweg, Ben A. W. de Jong, Harry E. Prince, Mary Lapé-Nixon, Pieter G. M. Gageldonk, Irina Tcherniaeva, Ingeborg Aaberge, Tove Karin Herstad, Merit Melin, Ger T. Rijkers, Guy A. Berbers

**Affiliations:** a Medical Microbiology & Immunology Department, St Antonius Hospital Nieuwegein, Nieuwegein, The Netherlands; b National Institute for Health and Welfare, Helsinki, Finland; c Statens Serum Institut, Copenhagen, Denmark; d Public Health England, Public Health Laboratory, Manchester, United Kingdom; e Public Health Agency of Sweden, Stockholm, Sweden; f Reinier HAGA MDC, Delft, The Netherlands; g National Institute of Public Health and the Environment (RIVM), Bilthoven, The Netherlands; h Quest Diagnostics Infectious Disease, San Juan Capistrano, California, USA; i Norwegian Institute of Public Health, Oslo, Norway; j University College Roosevelt, Middelburg, The Netherlands; U.S. Food and Drug Administration

**Keywords:** IgG antibodies, *Streptococcus pneumoniae*, capsular polysaccharide, concordance, immunoserology, interlaboratory comparison, multiplex immunoassay, quantitative methods, serosurveillance

## Abstract

Surveillance studies are required to estimate the impact of pneumococcal vaccination in both children and the elderly across Europe. The World Health Organization (WHO) recommends use of enzyme immunoassays (EIAs) as standard methods for immune surveillance of pneumococcal antibodies. However, as levels of antibodies to multiple serotypes are monitored in thousands of samples, a need for a less laborious and more flexible method has evolved. Fluorescent-bead-based multiplex immunoassays (MIAs) are suitable for this purpose. An increasing number of public health and diagnostic laboratories use MIAs, although the method is not standardized and no international quality assessment scheme exists. The EU Pneumo Multiplex Assay Consortium was initiated in 2013 to advance harmonization of MIAs and to create an international quality assessment scheme. In a multilaboratory comparison organized by the consortium, agreement among nine laboratories that used their own optimized MIA was assessed on a panel of 15 reference sera for 13 pneumococcal serotypes with the new WHO standard 007sp. Agreement was assessed in terms of assay accuracy, reproducibility, repeatability, precision, and bias. The results indicate that the evaluated MIAs are robust and reproducible for measurement of vaccine-induced antibody responses. However, some serotype-specific variability in the results was observed in comparisons of polysaccharides from different sources and of different conjugation methods, especially for serotype 4. On the basis of the results, the consortium has contributed to the harmonization of MIA protocols to improve reliability of immune surveillance of Streptococcus pneumoniae.

**IMPORTANCE** Serology of Streptococcus pneumoniae is challenging due to existence of multiple clinically relevant serotypes and the introduction of multivalent vaccines in national immunization programs. Multiplex immunoassays (MIAs) are applied as high-throughput cost-effective methods for serosurveillance, and yet laboratories use their own protocols. The aims of this study were to assess the agreement of results generated by MIAs in different laboratories within the EU Pneumo Multiplex Assay Consortium, to analyze factors contributing to differences in outcome, and to create a harmonized protocol. The study demonstrated good agreement of results of MIAs performed by laboratories using controlled assays for determination of levels of vaccine-induced pneumococcal antibodies. The EU Pneumo Multiplex Assay Consortium is open to everyone working in public health services, and it aims to facilitate efforts by participants to run and maintain a cost-effective, reproducible, high-quality MIA platform.

## Abstract (precis)

Serology of Streptococcus pneumoniae is challenging due to existence of multiple clinically relevant serotypes and the introduction of multivalent vaccines in national immunization programs. Multiplex immunoassays (MIAs) are applied as high-throughput cost-effective methods for serosurveillance, and yet laboratories use their own protocols. The aims of this study were to assess the agreement of results generated by MIAs in different laboratories within the EU Pneumo Multiplex Assay Consortium, to analyze factors contributing to differences in outcome, and to create a harmonized protocol. The study demonstrated good agreement of results of MIAs performed by laboratories using controlled assays for determination of levels of vaccine-induced pneumococcal antibodies. The EU Pneumo Multiplex Assay Consortium is open to everyone working in public health services, and it aims to facilitate efforts by participants to run and maintain a cost-effective, reproducible, high-quality MIA platform.

## INTRODUCTION

Streptococcus pneumoniae (the pneumococcus) is an encapsulated bacterium that causes major infections during childhood and in the elderly, including pneumonia, meningitis, otitis media, and sepsis (1–3). Worldwide, pneumococci are the cause of death of more than 1 million children each year, while older adults who are >65 years of age are disproportionately affected by pneumonia requiring hospital care (4–7). The prevalence of pneumococcal disease varies with age but also with ethnic background, geographical location, and time (8). A particular aspect of this bacterium is the existence of multiple serotypes (nearly 98) that differ by the chemical composition of their polysaccharide capsule (9, 10). About 25% of the serotypes are responsible for the majority of pneumococcal disease in a given region (11).

Protection against pneumococcal infections is mediated by the concerted action of antibodies and complement components opsonizing the bacteria for phagocytosis (12, 13). Serum antibodies against pneumococcal capsular polysaccharides (PPS) can protect against pneumococcal infection in a serotype-specific manner (12, 13). The licensed pneumococcal vaccines contain a mixture of polysaccharides of different serotypes (23-valent polysaccharide vaccine) or of polysaccharides of multiple serotypes conjugated to protein carriers (10-valent or 13-valent conjugate vaccines). Vaccination against pneumococcus has been introduced as a health care intervention in many countries (14).

Surveillance studies are required to estimate the nature and the impact of this health care intervention for both children and the elderly across Europe. For decades, the World Health Organization (WHO) has recommended the use of enzyme immunoassays (EIAs) as standard methods for immunosurveillance of anti-PPS antibodies. WHO has established standard reference sera for use in EIA (15–17). The first standard serum, 89SF, was replaced by a new standard serum, 007sp, in 2011 by bridging to the previous standard (17). As multiple serotypes have to be monitored and as surveillance studies usually involve thousands of samples, it has been recognized for some time that a less laborious and more flexible method would be preferable. Fluorescent-bead-based multiplex immunoassays (MIAs), in combination with detailed descriptions of chemical coupling techniques, have been developed in various laboratories and national institutions (18–20). This anti-PPS antibody screening method has been shown to be robust, time-efficient, automatable, and economically feasible.

The recognition of pneumococcus as an important pathogen to be monitored, along with the growing number of laboratories performing multiplex analysis of anti-PPS antibodies, resulted in the initiation of the EU Pneumo Multiplex Assay Consortium in 2013. The main goals of this nonprofit consortium are to share knowledge, analyze and harmonize MIA protocols, and offer an international quality assessment scheme. Participants include public health organizations and diagnostic laboratories as well as industry. So far, the consortium has organized 6 annual meetings in 5 different countries.

In this paper, we describe and discuss the results of interlaboratory comparisons in which 11 different laboratories participated using two different assay platforms, EIA and MIA, for determination of serum anti-PPS IgG antibodies. In addition, this paper represents the first multilaboratory study to use the new WHO standard, 007sp, for quantitation of pneumococcal antibodies. The study was conducted to primarily assess the level of agreement of the MIA among laboratories using their own protocols without the introduction of any common reagents other than the serum samples to be evaluated. Other aims were to assess the agreement between results obtained by MIA and those obtained by the standard WHO-approved EIA method using the new 007sp standard and, finally, to investigate in detail possible causes of variations in outcome according to the different sources of PPS and the different PPS conjugation methods used.

## RESULTS

### Interlaboratory agreement for IgG antibody concentrations.

Agreement between all participating laboratories was assessed between pairs of laboratories for data aggregated over serotypes and for serotype-specific data (Table 1). On the basis of the aggregated data, the laboratories generally performed comparably to each other for accuracy (Lin’s coefficient of accuracy [*C_a_*] range, 0.90 to 0.99), precision (i.e., the measure of how far a set of observations deviates from a straight line, quantified using Pearson’s correlation coefficient [*r*]) (range, 0.84 to 0.99), and concordance (Lin’s concordance correlation coefficient [*r_c_*], which represents a combination of *C_a_* and *r*) (range, 0.79 to 0.98). The *r_c_* values describing both accuracy and precision were >0.80 for 35/36 and ≥0.90 for 22/36 of the pairwise comparisons.

**TABLE 1:** Comparison of IgG concentrations determined by MIA between different laboratories and the assigned IgG antibody concentrations and serotype-specific comparison of IgG concentrations determined by MIA in different laboratories to the assigned IgG antibody concentrations^*a*^

Laboratory or serotype	Statistic	Values for laboratory:								
		I	II	III	IV	V	VI	VII	VIII	IX
Comparison of [IgG]										
I	*C_a_*	1.0	0.920	0.972	0.960	0.990	0.943	0.896	0.912	0.926
	*r*	1.0	0.904	0.854	0.895	0.853	0.840	0.907	0.881	0.900
	*r_c_*	1.0	0.831 (0.777–0.873)	0.830 (0.776–0.872)	0.860 (0.817–0.893)	0.844 (0.793–0.884)	0.792 (0.733–0.839)	0.812 (0.760–0.853)	0.804 (0.748–0.848)	0.833 (0.783–0.872)
II	*C_a_*		1.0	0.975	0.960	0.935	0.942	0.999	0.996	0.997
	*r*		1.0	0.964	0.982	0.940	0.948	0.978	0.970	0.980
	*r_c_*		1.0	0.940 (0.918–0.956)	0.942 (0.925–0.956)	0.879 (0.840–0.910)	0.893 (0.861–0.918)	0.977 (0.967–0.983)	0.965 (0.952–0.975)	0.977 (0.969–0.984)
III	*C_a_*			1.0	0.997	0.996	0.991	0.949	0.967	0.977
	*r*			1.0	0.942	0.906	0.906	0.954	0.918	0.959
	*r_c_*			1.0	0.940 (0.920–0.955)	0.903 (0.871–0.927)	0.898 (0.865–0.923)	0.906 (0.878–0.927)	0.888 (0.851–0.916)	0.936 (0.916–0.952)
IV	*C_a_*				1.0	0.987	0.997	0.941	0.965	0.971
	*r*				1.0	0.936	0.967	0.974	0.981	0.969
	*r_c_*				1.0	0.924 (0.901–0.942)	0.963 (0.951–0.973)	0.917 (0.895–0.934)	0.946 (0.931–0.959)	0.940 (0.923–0.954)
V	*C_a_*					1.0	0.972	0.955	0.954	0.977
	*r*					1.0	0.916	0.917	0.944	0.926
	*r_c_*					1.0	0.891 (0.860–0.915)	0.876 (0.838–0.905)	0.901 (0.870–0.925)	0.905 (0.874–0.928)
VI	*C_a_*						1.0	0.921	0.947	0.956
	*r*						1.0	0.936	0.964	0.937
	*r_c_*						1.0	0.862 (0.827–0.891)	0.913 (0.889–0.932)	0.896 (0.866–0.919)
VII	*C_a_*							1.0	0.995	0.994
	*r*							1.0	0.966	0.986
	*r_c_*							1.0	0.961 (0.948–0.971)	0.981 (0.974–0.985)
VIII	*C_a_*								1.0	0.999
	*r*								1.0	0.959
	*r_c_*								1.0	0.958 (0.943–0.969)
IX	*C_a_*									1.0
	*r*									1.0
	*r_c_*									1.0
										
Assigned values	*C_a_*	0.952	0.866	0.960	0.971	0.946	0.971	0.846	0.888	0.892
	*r*	0.847	0.930	0.881	0.921	0.878	0.882	0.917	0.921	0.925
	*r_c_*	0.806 (0.753–0.849)	0.806 (0.755–0.847)	0.846 (0.802–0.881)	0.894 (0.862–0.919)	0.831 (0.787–0.866)	0.856 (0.811–0.891)	0.776 (0.726–0.818)	0.818 (0.771–0.856)	0.825 (0.782–0.860)
										
Serotype-specific comparison of [IgG]										
1	*C_a_*	0.665	0.889	0.927	0.963	0.789	0.955	0.738	0.840	0.862
	*r*	0.871	0.966	0.977	0.963	0.956	0.961	0.939	0.917	0.957
	*r_c_*	0.579 (0.293–0.770)	0.859 (0.725–0.930)	0.906 (0.813–0.954)	0.927 (0.828–0.970)	0.755 (0.521–0.883)	0.918 (0.756–0.974)	0.693 (0.448–0.841)	0.771 (0.524–0.898)	0.824 (0.653–0.916)
3	*C_a_*	0.915	NA	0.714	0.999	0.821	0.901	0.764	0.686	0.762
	*r*	0.760	NA	0.816	0.770	0.763	0.591	0.760	0.666	0.785
	*r_c_*	0.696 (0.348–0.875)	NA	0.582 (0.252–0.791)	0.769 (0.404–0.923)	0.626 (0.243–0.840)	0.533 (0.067–0.808)	0.580 (0.218–0.802)	0.457 (0.086–0.717)	0.598 (0.250–0.810)
4	*C_a_*	0.842	0.683	0.801	0.920	0.849	0.845	0.703	0.806	0.782
	*r*	0.906	0.907	0.964	0.883	0.852	0.645	0.945	0.807	0.954
	*r_c_*	0.762 (0.488–0.900)	0.619 (0.332–0.802)	0.773 (0.559–0.890)	0.812 (0.539–0.931)	0.723 (0.470–0.866)	0.545 (0.150–0.790)	0.664 (0.403–0.826)	0.651 (0.294–0.848)	0.746 (0.506–0.879)
5	*C_a_*	0.948	0.893	0.967	0.979	0.798	0.947	0.935	0.934	0.960
	*r*	0.967	0.916	0.956	0.966	0.962	0.925	0.949	0.950	0.965
	*r_c_*	0.916 (0.793–0.967)	0.818 (0.620–0.918)	0.924 (0.797–0.973)	0.945 (0.861–0.979)	0.768 (0.547–0.889)	0.875 (0.672–0.956)	0.887 (0.727–0.955)	0.888 (0.721–0.957)	0.927 (0.832–0.969)
6A	*C_a_*	NA	NA	0.784	0.879	0.454	0.835	0.675	NA	0.744
	*r*	NA	NA	0.747	0.728	0.723	0.720	0.706	NA	0.758
	*r_c_*	NA	NA	0.586 (0.214–0.809)	0.640 (0.237–0.855)	0.328 (0.06–0.552)	0.601 (0.204–0.829)	0.476 (0.116–0.726)	NA	0.563 (0.200–0.791)
6B	*C_a_*	0.749	0.730	0.885	0.902	0.890	0.907	0.702	0.798	0.761
	*r*	0.881	0.920	0.940	0.933	0.933	0.913	0.925	0.933	0.937
	*r_c_*	0.660 (0.356–0.838)	0.672 (0.405–0.833)	0.832 (0.660–0.921)	0.842 (0.648–0.933)	0.830 (0.638–0.925)	0.828 (0.608–0.930)	0.649 (0.382–0.816)	0.745 (0.511–0.876)	0.714 (0.470–0.856)
7F	*C_a_*	0.762	0.759	0.963	0.925	0.996	0.857	0.893	0.833	0.876
	*r*	0.940	0.916	0.959	0.964	0.976	0.948	0.979	0.971	0.963
	*r_c_*	0.716 (0.454–0.864)	0.696 (0.416–0.855)	0.924 (0.796–0.973)	0.892 (0.735–0.958)	0.972 (0.914–0.991)	0.812 (0.589–0.920)	0.874 (0.721–0.946)	0.809 (0.606–0.913)	0.844 (0.654–0.934)
9V	*C_a_*	0.943	0.727	0.851	0.871	0.924	0.844	0.645	0.733	0.724
	*r*	0.867	0.902	0.941	0.890	0.928	0.897	0.891	0.904	0.912
	*r_c_*	0.817 (0.560–0.930)	0.655 (0.368–0.828)	0.801 (0.586–0.911)	0.776 (0.500–0.909)	0.857 (0.674–0.941)	0.793 (0.525–0.918)	0.575 (0.285–0.768)	0.663 (0.384–0.831)	0.660 (0.382–0.829)
14	*C_a_*	0.960	0.888	0.898	0.960	0.935	0.967	0.882	0.936	0.904
	*r*	0.959	0.969	0.965	0.977	0.976	0.935	0.974	0.985	0.931
	*r_c_*	0.921 (0.827–0.965)	0.860 (0.752–0.924)	0.867 (0.706–0.943)	0.938 (0.876–0.970)	0.913 (0.832–0.956)	0.905 (0.739–0.967)	0.859 (0.746–0.923)	0.922 (0.835–0.964)	0.841 (0.665–0.929)
18C	*C_a_*	0.959	0.884	0.970	0.954	0.885	0.970	0.853	0.860	0.911
	*r*	0.922	0.939	0.968	0.934	0.937	0.910	0.934	0.941	0.945
	*r_c_*	0.884 (0.688.0.969)	0.830 (0.618–0.929)	0.939 (0.844–0.977)	0.891 (0.714–0.961)	0.829 (0.610–0.931)	0.883 (0.677–0.961)	0.797 (0.567–0.912)	0.808 (0.583–0.918)	0.861 (0.676–0.944)
19A	*C_a_*	0.767	NA	0.871	0.927	0.874	0.922	0.772	0.785	0.821
	*r*	0.622	NA	0.778	0.819	0.881	0.838	0.826	0.824	0.812
	*r_c_*	0.477 (0.07–0.747)	NA	0.678 (0.309–0.869)	0.759 (0.441–0.908)	0.770 (0.513–0.900)	0.773 (0.463–0.914)	0.637 (0.331–0.822)	0.646 (0.327–0.833)	0.667 (0.335–0.852)
19F	*C_a_*	0.765	0.881	0.872	0.979	0.949	0.971	0.848	0.900	0.914
	*r*	0.925	0.983	0.991	0.979	0.983	0.978	0.981	0.968	0.979
	*r_c_*	0.708 (0.435–0.862)	0.866 (0.737–0.934)	0.863 (0.724–0.935)	0.958 (0.902–0.982)	0.933 (0.855–0.969)	0.950 (0.874–0.981)	0.831 (0.676–0.916)	0.871 (0.749–0.936)	0.895 (0.778–0.952)
23F	*C_a_*	0.978	0.879	0.911	0.979	0.895	0.949	0.808	0.899	0.883
	*r*	0.861	0.862	0.932	0.843	0.906	0.893	0.865	0.896	0.899
	*r_c_*	0.842 (0.593–0.944)	0.758 (0.473–0.899)	0.849 (0.662–0.936)	0.825 (0.537–0.941)	0.811 (0.562–0.925)	0.848 (0.603–0.947)	0.699 (0.406–0.862)	0.805 (0.551–0.922)	0.794 (0.555–0.911)
										
Total	*C_a_*	0.952	0.866	0.960	0.971	0.946	0.971	0.846	0.888	0.892
	*r*	0.847	0.930	0.881	0.921	0.878	0.882	0.917	0.921	0.925
	*r_c_*	0.806 (0.753–0.849)	0.806 (0.755–0.847)	0.846 (0.802–0.881)	0.894 (0.862–0.919)	0.831 (0.787–0.866)	0.856 (0.811–0.891)	0.776 (0.726–0.818)	0.818 (0.771–0.856)	0.825 (0.782–0.860)

a *C_a_*, accuracy; *r*, precision; *r_c_*, concordance correlation coefficient; NA, not available; [IgG], IgG concentration. Values in parenthesis are 95% confidence intervals. Assigned values are the WHO-assigned IgG concentrations for 12 pneumococcal calibration sera as determined with the pneumococcal standard 007sp (17).

**FIG 1:**
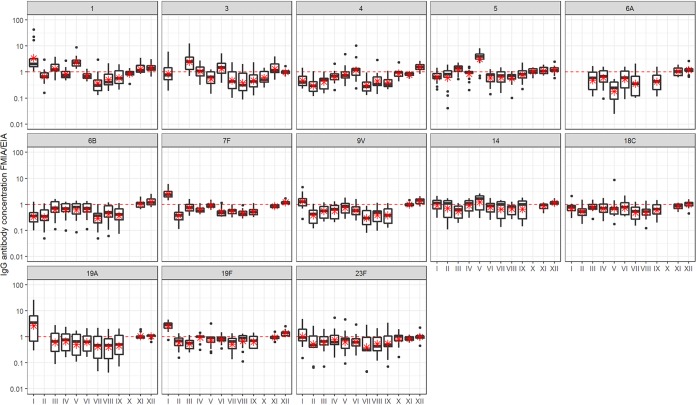
Box plots by serotype and laboratory (I to XII) for ratios of IgG antibody concentrations (micrograms per milliliter) determined by MIA or EIA to the assigned IgG concentrations by WHO. The hinges and the horizontal line of the box represent the 25th and 75th percentiles and the median, respectively, and the red asterisks signify means. The size of the box, coupled with the whiskers, is a direct indicator of the within-laboratory variability of the bias corresponding to the MIA/EIA ratios. The circles correspond to outlying assay values. The positioning of the boxes around the dotted, ideal line with a coefficient of 1 for a given serotype across all laboratories is an indicator of the between-laboratory variability. Results from laboratories I to IX were determined by MIA and results from laboratories X, XI, and XII by the WHO EIA. The number of samples analyzed per serotype was 13.

The degree of agreement did, however, vary by laboratory and serotype. Laboratory I consistently overestimated the antibody concentrations for serotypes 1, 7F, 19A, and 19F and laboratory V the antibody concentrations for serotypes 1 and 5 compared to the antibody concentrations observed in the other laboratories, as illustrated in Fig. 1. This is also seen in the serotype-specific concordance between laboratories I and V and the other laboratories for these serotypes; between laboratory I and the other laboratories, *r_c_* ranged between 0.45 and 0.77 for serotypes 1, 7F, 19A, and 19F, and between laboratory V and the other laboratories, *r_c_* ranged between 0.58 and 0.91 for serotypes 1 and 5. In the pairwise comparisons between other laboratories, ≥95% yielded *r_c_* values of >0.80 for these serotypes. For all of the other serotypes, the percentages of pairwise laboratory-to-laboratory comparisons yielding *r_c_* values of >0.8 were 39%, 50%, 80%, 100%, 86%, 100%, 100%, and 100% for serotypes 3, 4, 6A, 6B, 9V, 14, 18C, and 23F, respectively. In line with this, the variation of the ratio of observed IgG concentrations to assigned values between laboratories, illustrated by the positioning of the boxes about the dotted line in Fig. 1, was clearly dependent on the serotype, being lowest for serotypes 14, 18C, and 23F and greatest for serotypes 1, 3, and 4 (Fig. 1).

### Agreement between MIA and the assigned values for IgG concentrations.

The serotype-specific IgG antibody concentrations determined using the new 007sp standard have been assigned for a panel of 12 WHO calibration sera for 13 serotypes by EIA (17). Box plots displaying the distribution of MIA/EIA ratios between the individual laboratory-reported concentrations (MIA) and the assigned IgG concentrations (EIA) are presented by serotype in Fig. 1 (laboratories I to IX). The data from most laboratories reporting results by MIA had a negative mean bias for the majority of the serotypes analyzed, meaning that the IgG concentrations measured by MIA are in general lower than the assigned values. Two laboratories (I and V), however, had a substantial positive bias for 5/13 and 2/13 serotypes. All laboratories showed only a minor bias for serotype 14.

Overall, the within-laboratory variability of the bias among the samples was higher in MIA than in the standardized WHO EIA for all serotypes (Fig. 1). Serotypes 3, 6A, and 19A showed the highest within-laboratory variability of the bias between the IgG concentration measured by MIA and the assigned antibody concentration (Fig. 1). For serotypes 6A and 19A, the mean bias was negative for most laboratories, but the bias was positive for serotype 3 in three laboratories and negative in four laboratories, and one laboratory showed no bias. The agreement between the laboratories (X, XI, and XII) reporting IgG concentrations determined by EIA was excellent, showing practically no bias toward the assigned IgG concentrations. Assessing within-laboratory variation by coefficients of variation calculated from triplicate measurements (or from duplicate measurements for laboratory II) within each laboratory, the highest variation was seen for serotypes 19A, 7F, and 5 (see Fig. S1 in the supplemental material).

10.1128/mSphere.00455-19.2 FIG S1 Within-laboratory variability (CV%) of MIA by PPS serotype. *n* = 13 to 15 depending on laboratory and serotype. Download FIG S1, TIF file, 0.1 MB. Copyright © 2019 Meek et al. 2019 Meek et al. This content is distributed under the terms of the Creative Commons Attribution 4.0 International license.

Despite the observed negative bias, the assessed correlation between the IgG concentrations measured by MIA in each laboratory and the assigned IgG antibody concentrations was good for data aggregated over serotypes (*r *= 0.85 to 0.93, depending on laboratory) (Table 1, comparison of IgG concentrations determined by MIA) and for the serotype-specific data (generally, *r* = 0.85 to 0.99, excluding serotype 4 for laboratories VI and VIII), with the exception of serotypes 3, 6A, and 19A, which showed poorer correlation (*r* = 0.59 to 0.88, depending on the laboratory) (Table 1, serotype-specific comparison). Similarly, the serotype-specific analysis of concordance showed serotype-specific differences between IgG concentrations observed by MIA and the assigned values (Table 1, serotype-specific comparison of IgG concentrations determined by MIA). The *r_c_* values generally ranged between 0.50 and 0.95 for different serotypes and laboratories. The lowest *r_c_* values were observed for serotypes 6A, 3, 19A, and 4 (*r_c_* range, 0.33 to 0.77 for all laboratories, with the exception of laboratory IV for serotype 4) and the highest for serotypes 14, 5, 18C, 19F, and 7F (*r_c_* range of 0.70 to 0.97 and *r_c_* of ≥0.80 for all laboratories except for laboratory V for serotype 5, laboratories I and II for serotype 7F, and laboratory I for serotype 19F) (Table 1, serotype-specific comparison of IgG concentrations determined by MIA).

**FIG 2:**
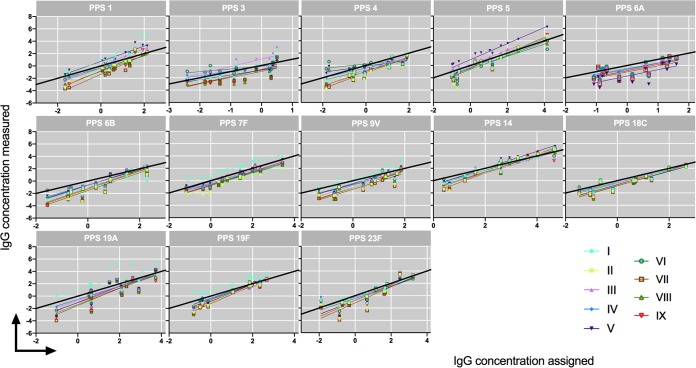
Linear regression analysis panels for each serotype, with a different line for each laboratory (I to IX). In total, 13 samples with assigned concentrations were tested: 12 sera from WHO panel B and sample 007sp. Each dot represents the mean of results of 2 or 3 measurements of each sample. The color code of each laboratory is indicated. The line of identity is marked in black. Corresponding data representing outcomes of the linear regression analysis (slope/confidence intervals) can be found in Table S2. Laboratory II did not submit data for serotype 3 and 19A, and laboratories I to II and VIII did not submit data for serotype 6A. All values are in micrograms per milliliter (log transformed).

Plotting assigned values versus outcome for each sample per serotype and per laboratory showed that most linear regression lines appeared close to or below the line of identity (Fig. 2). This confirms the overall negative mean bias observed already (Fig. 1). In general, regression lines had slopes of >1 and approached the line of identity when sera contained higher concentrations of serotype-specific antibodies. Most slopes were categorized as representing good to excellent results. Several exceptions were noted. Laboratories VII, VIII, and IX showed good precision for serotype 6B (*r* range, 0.93 to 0.94), and yet the slopes of the fitted regression line were somewhat steeper (slope = 1.5) (see Table S1 in the supplemental material), affecting the concordances (*r_c_* range, 0.65 to 0.75) (Fig. 2 and Table 1, serotype-specific comparison of IgG concentrations determined by MIA). A similar combination of slope and precision was found for laboratories II and VII regarding serotype 14, and yet their overall concordance was good (*r_c_* values of >0.8) (Fig. 2; see also Table S2 and Table 1, serotype-specific comparison of IgG concentrations determined by MIA). A second type of exception was represented by regression lines above line of identity. This was observed for laboratory I for serotypes 7F, 19A, and 19F and for laboratory V for serotype 5 (Fig. 2). The slopes of the linear regression lines were categorized as excellent for these serotypes, with good precision, except for 19A in the case of laboratory I (*r* = 0.62), and yet affected the overall concordance of the data from laboratory I for serotypes 7F, 19A, and 19F (*r_c_* = 0.72, 0.48, and 0.71, respectively) (Table 1, serotype-specific comparison of IgG concentrations determined by MIA) (Fig. 2; see also Table S2). Other exceptions were noted for serotypes 3, 4, and 6A. For serotype 3 and 4, regression lines were divergent between laboratories. Concerning serotype 3, the data from laboratory VI in particular showed poor agreement regarding the slope (slope = 0.44), along with poor concordance (*r_c_* = 0.53) (Table 1, comparison of IgG concentration ratios determined by MIA) (Fig. 2; see also Table S1). The data from most other laboratories had poor concordances for this serotype as well (*r_c_* = 0.46 to 0.77) (Table 1, serotype-specific comparison of IgG concentrations determined by MIA). Only the data from laboratory III displayed both an excellent slope and good precision (slope = 1.2, *r_c_* = 0.82), but the regression line ran well above the line of identity, resulting in poor concordance (*r_c_* = 0.58) (Table 1, serotype-specific comparison of IgG concentrations determined by MIA) (Fig. 2; see also Table S2). With serotype 4, the data from laboratories V and VI had regression slopes that were poor (slope = 0.50 for lab V and 0.37 for lab VI), together with poor concordance in the case of laboratory VI (*r_c_* = 0.55) (Table 1, serotype-specific comparison of IgG concentrations determined by MIA) (Fig. 2; see also Table S2). Regarding serotype 6A, there was good agreement with respect to the slopes, but the absolute concentrations measured by each laboratory differed considerably (Table S2) (Fig. 2), resulting in overall poor concordances (*r_c_* = 0.33 to 0.64). Not all laboratories (6/9) had this serotype included in their panel.

10.1128/mSphere.00455-19.6 TABLE S1 Overview of MIA protocols used by participating laboratories. ADHS, antibody-depleted human serum; ATCC, American Type Culture Collection; CWPS, cell wall polysaccharide; DMTMM, 4-(4,6-dimethoxy-1,3,5-triazin-2-yl)-4-methyl-morpholinium chloride; EDC, 1-ethyl-3-(3-dimethylaminopropyl)carbodiimide; FBS, fetal bovine serum; NBBS, newborn bovine serum; NIBSC, National Institute for Biological Standards and Control; ON, overnight; PBS, phosphate-buffered saline; PLL, poly-l-lysine; PPS, pneumococcal polysaccharide; RPE, phycoerythrin; RT, room temperature; SSI, Statens Serum Institut; Sulpho-NHS, sulpho-n-hydroxysuccinimide; w/o, without. Download Table S1, DOCX file, 0.05 MB. Copyright © 2019 Meek et al. 2019 Meek et al. This content is distributed under the terms of the Creative Commons Attribution 4.0 International license.

10.1128/mSphere.00455-19.7 TABLE S2 Support for Fig. 2. Data represent linear regression slopes (with 95% confidence intervals) per serotype for each laboratory. Download Table S2, DOCX file, 0.1 MB. Copyright © 2019 Meek et al. 2019 Meek et al. This content is distributed under the terms of the Creative Commons Attribution 4.0 International license.

### Analysis of variables affecting interlaboratory reproducibility.

Per study design, all participating laboratories used their own MIA, with no common reagents other than the samples to be evaluated. For analysis of variables possibly affecting the assay reproducibility, all laboratories were advised to report the source and batch numbers of the pneumococcal capsular polysaccharide products and the bead conjugation method used (see Table S1 in the supplemental material).

10.1128/mSphere.00455-19.1 TEXT S1 Batch numbers of ATCC and SSI Diagnostica polysaccharides used for comparative analysis shown in Fig. S2 and S3. Download Text S1, DOCX file, 0.01 MB. Copyright © 2019 Meek et al. 2019 Meek et al. This content is distributed under the terms of the Creative Commons Attribution 4.0 International license.

### Comparison of polysaccharides from two different sources.

In general, the comparison of IgG antibody concentrations obtained using polysaccharides from ATCC or SSI Diagnostica displayed good to excellent agreement (slope = 0.73 to 1.15, *r* = 0.80 to 0.99, depending on the serotype) (Table 2), with the exception of the serotype 3 data, which showed poor agreement (slope = 0.54, *r* = 0.66) (Table 2). Two serotypes also stood out by having only good agreement: 6B and 19F (slope = 0.73 and 0.78, *r* = 0.88 and 0.80) (Table 2). The slope for serotype 1 was excellent; the wide confidence intervals (0.49 to 1.30) did not, however, indicate a strong linear relationship. For serotypes 1 and 6B, quantitation using polysaccharides from SSI Diagnostica resulted in slightly higher concentrations for sera with low IgG values. However, interpretation of results obtained for PPS 3, 6B, and 19F should be performed with care, as most data points were aggregated, causing outliers to have a strong impact on the correlation analysis (see Fig. S2 in the supplemental material).

**TABLE 2:** Deming regression and Pearson correlation per serotype for quantitation of anti-PPS antibodies in 13 sera from WHO panel B determined using MIA beads coated with polysaccharides obtained from ATCC or SSI Diagnostica^*a*^

PPS	Slope	LCI	UCI	*r*
1	0.99	0.49	1.30	0.87
3	0.54	0.35	0.77	0.66
4	0.99	0.85	1.12	0.93
5	1.12	0.99	1.35	0.95
6B	0.73	0.45	0.82	0.88
7F	1.06	1.01	1.12	0.99
9V	1.04	0.89	1.16	0.94
14	0.94	0.80	1.06	0.95
18C	1.15	1.03	1.27	0.97
19A	0.99	0.88	1.12	0.97
19F	0.78	0.51	0.98	0.80
23F	0.94	0.87	1.00	0.99

a LCI, lower confidence interval value; UCI, upper confidence interval value.

Compared with the assigned values for WHO serum panel B, most polysaccharides from either source yielded good or excellent agreement between the quantitated and assigned values (slope = 0.84 to 1.39 and 0.69 to 1.15 and *r* = 0.75 to 0.97 and 0.78 to 0.98, respectively, for PPS originating from ATCC and SSI Diagnostica) (Table 3; see also Fig. S2), with the exception of particular serotypes, namely, serotype 6B (ATCC and SSI Diagnostica) (slope = 0.59 and 0.34 and *r* = 0.87 and 0.69, respectively), serotype 19A (ATCC and SSI Diagnostica) (slope = 0.89 and 0.73, respectively, and *r* = 0.66 to 0.60 for both), serotype 14 (ATCC) (slope = 1.48 and *r* = 0.94), and serotype 19F (SSI Diagnostica) (slope = 0.42 and *r* = 0.65) (Table 3). It was striking that serotype 19A performed well in the comparisons of PPSs from different sources (slope = 0.96, *r* = 0.97) and yet these results clearly deviated from the assigned values (slope = 0.89 and 0.73 and *r* = 0.66 and 0.60 for PPS from ATCC and SSI Diagnostica, respectively) (Table 3). This may have been assay related, as the antibodies to two cross-reacting serotypes, 19A and 19F, were quantitated simultaneously in this particular MIA setting. In EIA, in contrast, antibodies to each serotype are quantitated in separate assays. A general potential bias in the comparison is that all of the values assigned for WHO serum panels A and B have been determined using polysaccharides from ATCC. Regarding repeatability (or precision), quantitation done with PPS from either source proved to be highly repeatable (slope = 0.87 to 1.1 and *r* = 0.98 to 1.00, depending on the serotype) (Fig. S3).

**TABLE 3:** Slope and Pearson correlation coefficient values for calculated and WHO-assigned values using MIA with ATCC polysaccharides or SSI Diagnostica polysaccharides

PPS	ATCC		SSI Diagnostica	
	Slope	*r*	Slope	*r*
1	1.39	0.96	1	0.78
3	0.98	0.75	0.78	0.85
4	0.85	0.91	0.69	0.85
5	0.97	0.97	1	0.93
6B	0.59	0.87	0.34	0.69
7F	0.90	0.96	0.96	0.98
9V	1.06	0.90	0.97	0.90
14	1.48	0.94	1.15	0.87
18C	1.03	0.94	1.08	0.89
19A	0.89	0.66	0.73	0.60
19F	0.84	0.94	0.42	0.65
23F	1.06	0.83	0.96	0.84

10.1128/mSphere.00455-19.3 FIG S2 Linear regression analysis of results of comparisons between calculated and assigned antipneumococcus (anti-Pn) concentration from WHO panel B, using polysaccharides from ATCC (left panel) or SSI Diagnostica (right panel). The blue line shows linear regression, and the gray area surrounding the blue line represents the 95% CI for the linear regression. Red dotted lines indicate 95% CI for the predicted model of the linear regression. All values represent micrograms per milliliter (log transformed). Download FIG S2, TIF file, 1.9 MB. Copyright © 2019 Meek et al. 2019 Meek et al. This content is distributed under the terms of the Creative Commons Attribution 4.0 International license.

10.1128/mSphere.00455-19.4 FIG S3 Reproducibility data obtained for ATCC and SSI Diagnostica polysaccharides. All values represent micrograms per milliliter (log transformed). Download FIG S3, TIF file, 1.8 MB. Copyright © 2019 Meek et al. 2019 Meek et al. This content is distributed under the terms of the Creative Commons Attribution 4.0 International license.

### Comparison of conjugation methods.

Two basic conjugation chemistries were employed by the participating laboratories to conjugate PPS to carboxylated beads in MIA: the two-step carbodiimide method involving poly-l-lysine (PLL) (18) and the method employing 4-(4,6-dimethoxy-1,3,5-triazin-2-yl)-4-methyl-morpholinium chloride (DMTMM) (20). The conjugation chemistry used by each participating laboratory is described in Table S1. Five laboratories used the PLL method, and four laboratories conjugated the beads using the DMTMM method. Overall, there was more variation in measured concentrations between PLL laboratories than between those using the DMTMM method. This was mainly caused by higher IgG concentrations measured by laboratories I and V for serotypes 1, 5, 7F, 19A, and 19F (Fig. 2). Furthermore, notable variability was seen between laboratories in the data for serotypes 1, 3, and 4. This considerable variability precludes an overall assessment of the contribution of the conjugation method to the interlaboratory variability in this study. A higher calculated antibody concentration (bias) was found only for serotype 4 conjugated by the PLL method for samples with relatively low specific IgG antibody concentrations against this serotype (slope = 0.59, *r* = 0.89) (Fig. S4).

10.1128/mSphere.00455-19.5 FIG S4 (A) Average outcomes for serotype 4 per conjugation method for each sample in calibration serum panel B and 007sp. Whiskers represent ± standard deviations (SD). (B) Deming regression analysis (slope = 0.57; 95% confidence interval, 0.34 to 0.81). All values represent micrograms per milliliter (log transformed). Download FIG S4, TIF file, 0.2 MB. Copyright © 2019 Meek et al. 2019 Meek et al. This content is distributed under the terms of the Creative Commons Attribution 4.0 International license.

## DISCUSSION

This study evaluated the level of agreement of nine laboratories for the quantitation of antipneumococcal capsular polysaccharide IgG antibodies by MIA. The laboratories used their own optimized assays without any common reagents other than the serum samples to be evaluated. The use of WHO enzyme-linked immunosorbent assay (ELISA) calibration serum panel B with assigned values provided a common reference and additionally enabled assessment of agreement between the data from each laboratory and the standardized EIA. The results obtained by MIA showed remarkably high agreement between laboratories: *r_c_* values of ≥0.79 for all pairwise comparisons of data aggregated over serotypes. In general, the results obtained by MIA correlated well with the WHO-assigned values, but a higher level of agreement was found between results obtained by MIA in different laboratories than between the MIA and WHO-assigned values.

The WHO recommends the use of the standardized enzyme immunoassay (EIA) as the primary method for evaluating serological responses to pneumococcal vaccines in infants. Alternative methods have to be bridged to this method to maintain the link with the pivotal clinical protection studies carried out during the licensure of the first conjugate vaccine (21). As multiple serotypes need to be monitored in surveillance studies of pneumococcal immunity that usually involve thousands of samples, many laboratories and national institutes have switched to high-throughput multiplex immunoassays in recent years. These assays are, however, not standardized, and no harmonized protocol has been available. As an approach to interlaboratory quality assurance for MIA, the interlaboratory agreement of MIA results has been assessed in three studies previously (22–24). In the study by Whaley et al., agreement of IgG concentrations determined by three bead-based immunoassays for WHO calibration serum panel B was assessed. The three assays showed modest (42% to 55%) agreement with the WHO-assigned values, with various levels of correlation between serotypes. The results of our study are in line with the results of that previous study in that higher interlaboratory agreement was observed between different MIAs than between MIA and the WHO-assigned values. Additionally, the concordance level of MIAs between laboratories in our study was good and was comparable to that reported by Whaley et al.; in 35 of 36 (97%) pairwise comparisons, the *r_c_* value was ≥0.80 (22). The studies of Zhang et al. and Daly et al. further evaluated whether the observed analytical variability between laboratories would affect the clinical classification of patients and responses by the use of paired clinical sera and published clinical algorithms (23, 24). They concluded that despite substantial variation seen in absolute values of data determined for pneumococcal antibodies, the overall classifications of the pneumococcal immune status of patients were remarkably similar between assays. The use of the WHO ELISA calibration sera in our study allowed us to evaluate only the absolute values of data from pneumococcal antibodies in those samples. What is similar between the current study and the earlier MIA comparison is that the within-laboratory variability is dependent on serotype. Our study employed a more comprehensive evaluation of bead-based pneumococcal assays than the previous studies and involved more laboratories than previously published in the literature. Furthermore, what distinguishes our efforts is that we have combined this with an analysis of factors that may have been responsible for the variation observed between laboratories.

Despite the relatively good overall agreement in MIA between laboratories, the level of agreement of MIA-assigned values varied by laboratory and serotype. Two laboratories (I and V) clearly deviated from the other laboratories regarding overall agreement and variability of MIA-assigned values. This was particularly reflected in the data determined for serotypes 1, 5, 7F, 19A, and 19F. The serotypes showing most variability across all laboratories were serotypes 1 and 3. Nevertheless, individual laboratories all showed good intralaboratory agreement (for the majority of laboratories and serotypes, the coefficient of variation [CV] was <15%).

To determine which factors may be responsible for poor concordances between laboratories, we have analyzed whether polysaccharide source and/or conjugation procedure can be held accountable. Next to serotypes 1 and 3, considerable variation between laboratories has been found for serotypes 4 and 6A as well. We have been able to rule out source of polysaccharides for serotypes 1 and 4 in that respect. Regarding serotype 1, it is still possible that differences in source and lot number contribute to the variation, but it must be a combination of (i) factors related to differences in details regarding conjugation procedures for polysaccharides to beads, (ii) shelf life of the bead-polysaccharide combinations used, and (iii) differences in measurement protocols that may have more effect on particular polysaccharides. Our results show that source of polysaccharides does have an effect on quantitation of serotype 3-specific antibodies. Even though not many laboratories used serotype 3 obtained from SSI, it will be interest to analyze different lots available through ATCC. At the same time, the clinical relevance of determining serotype 3-specific antibodies is being questioned (25), and inclusion of this serotype in MIA panels may become obsolete for laboratories that focus on protection and prevention of disease. Regarding serotype 4, this seems to be the only serotype for which conjugation procedure has differential effects on antigenicity. This phenomenon was not reported in the original publication (20). It will be worthwhile to have both conjugation procedures and shelf life of either conjugated polysaccharide-bead combination analyzed within one laboratory. Considering serotype 6A, we have not analyzed source and lot number differences or effects of differences in measurement protocols. It may be that some members within serotype families are more affected by differences in measurement protocols than others. The best way to resolve this issue, and all other sources of variation that we were unable to allocate, would be to share one batch of bead-polysaccharides and a serum panel between laboratories and analyze outcome. This would definitely help outlier laboratories to resolve their potential issues.

When results obtained by MIA from each laboratory were compared to the assigned IgG antibody values determined by the standardized WHO EIA, an overall negative bias was observed for most serotypes in the majority of laboratories, meaning that antibody levels generally are underestimated using MIA compared with WHO MIA. This was particularly observed at lower (<1 μg/ml) IgG antibody concentrations. Exceptions were already-mentioned serotypes 1 and 3, showing most variation and both negative and positive bias across laboratories. Another notable exception was serotype 14, for which only a minimal negative bias was observed in all laboratories, resulting in excellent agreement both between laboratories and between MIA and EIA.

Several factors can contribute to the observed bias. These may include different binding kinetics of antibodies to PPS adsorbed on solid phase or PPS on beads in liquid phase, conformational change in PPS as a result of the covalent coupling techniques, multiplexing itself, or differences in antigenicity of polysaccharide lots. In EIA the antibodies bind to excess of PPS adsorbed on solid surfaces of the wells of microtiter plates, whereas in MIA the antibodies bind to PPS on the surfaces of microspheres in suspension. It may be that higher relative avidity is required for binding to PPS conjugated to beads in MIA whereas high-avidity and low-avidity as well as cross-reactive and unspecific antibodies may be able to bind in EIA. The higher density of PPS on EIA plates than on the beads in MIA may favor the binding of both low-avidity and high-avidity antibodies. Preference of antibodies with high avidity could be one explanation for the negative bias of MIA results in comparison to WHO-assigned values. One may, however, speculate that the concentration of high-avidity antibodies is of most clinical importance upon encounter with PPS on the surface of bacteria. To elucidate this, evaluation using well-controlled avidity assays on lower-performance serotypes would be an important area of future research. To conclude, the negative mean bias between the results obtained by MIA and the assigned values will probably not have a major impact on conclusions drawn in surveillance studies assessing vaccine-induced antibodies or in evaluation of individual vaccine responses (with a focus on fold increase), which are the types of studies done by the laboratories within the consortium. Furthermore, underestimation of antibody concentration by a given assay or for an individual serotype may not affect clinical outcome, in contrast to overestimation of the concentration.

Several different methods for conjugation of beads with PPS have already been described and their comparison documented (19, 20, 26). Therefore, and since the primary aim of this study was to evaluate the level of agreement of MIA in laboratories using their own optimized assays without any common procedures or reagents, comparison of conjugation methods was not within the scope of this study. Due to several other variables affecting MIA performance, e.g., the use of different sources and batches of PPS in different laboratories and of different combinations of serotypes in assays, interpretation of results of conjugation method comparisons should be done with care. In general, comparable results were obtained regardless of conjugation method, with the exception of the results obtained with serotype 4.

Regarding polysaccharide lots, a high degree of agreement was found in IgG concentrations between PPS batches obtained from either ATCC or SSI Diagnostica, specifically, for serotypes 5, 7F, 18C, and 23F. In contrast, lower agreement was found for serotypes 3, 6B, and 19F. Understanding the effects of different polysaccharide batches may have in MIA will require extensive biochemical comparisons. Differential effects of purification procedures on labile groups attached to polysaccharides, such as O-acetylation, have been described previously (9). Such labile groups can be important for conformation and antigenicity of polysaccharides (9). Why a relatively simple polysaccharide such as a serotype 3 polysaccharide shows considerable heterogeneity between manufacturers, or between laboratories, for that matter, is not clear, and resolution of that issue would require additional biochemical and structural analysis. Because PPS 3 has a relatively simple configuration (9), the likelihood of loss of important epitopes in the conjugation procedure is increased, which may introduce more variability. Furthermore, various PPS batches obtained from different manufacturers have been previously compared using the EIA methodology (27, 28). Remarkable differences in antibody measurements using preparations from different manufacturers were reported, which eventually led to the inclusion of an irrelevant PPS 22F absorption step in EIA and later in MIA (29).

Our study had some limitations. The serum panel used for assessing agreement between laboratories and methods consisted solely of postvaccination adult sera with relatively high antipneumococcal IgG concentrations, and the number of samples assessed by serotype was relatively low. Hence, assessments of fold changes in antibody levels and, furthermore, clinical classification of responses were not possible with this sample panel. However, the ranges of the IgG concentrations in the WHO serum panel covered well the expected range in samples during immunosurveillance (30), except for serotype 14, for which assay comparability could be assessed only at IgG concentrations above 1.49 μg/ml.

The results of this study suggest that the bead-based multiplex immunoassays evaluated are robust and reproducible for the determination of vaccine-induced pneumococcal antibodies for immunosurveillance when performed by laboratories using controlled assays. However, we recognize that the assay did not perform optimally for all serotypes in all laboratories. On the basis of the results of this study and recent work of the consortium, a harmonized protocol for determination of pneumococcal antibodies by MIA has been created by the consortium. Individual laboratories have the opportunity to compare and improve performance regarding overall outcome, although it is not always evident what factor is responsible for a poorer outcome of a limited number of polysaccharides while most other serotypes perform well in the interlaboratory comparison.

On the basis of the results of this interlaboratory study, an important recommendation from the consortium for the PPS manufacturers is to supply more-detailed information regarding the composition and purity of the PPSs. The consortium and the PPS manufacturers could collaborate to establish methods to improve characterization and quality of PPS used in clinical diagnostic laboratories. Other future focus points of the consortium are to further harmonize the MIA protocols in order to improve accuracy and reproducibility on a Europe-wide scale. This would include efforts to create a shared platform for production of beads and to establish an expiration date for each bead-polysaccharide conjugate, particularly for PPS conjugated using DMTMM methodology. Furthermore, any laboratory using new platforms that allow multiplex analysis of anti-PPS antibodies would be invited to join the consortium, participate in the development of the quality assessment scheme, discuss outcome, and further improve on protocols. A better understanding of variables affecting variation between laboratories is likely to improve concordance with the WHO EIA. Collaboration will aim at improving the reliability of pan-European immunosurveillance of Streptococcus pneumoniae.

An important area of future evaluation by the pneumococcal assay scientific community will be the emerging replacement serotypes. The flexibility of MIA allows additional serotypes to be incorporated easily in a single assay as long as antibody values are assigned with respect to the reference serum. Future testing demands can be met by fully utilizing the capacity of the MIA-based techniques.

## MATERIALS AND METHODS

### Study design.

Eleven participating laboratories (listed in Table 4) measured IgG pneumococcal antibody concentrations in a panel of 15 samples distributed by Public Health England (PHE), United Kingdom. The panel consisted of 12 sera of pneumococcal ELISA calibration serum panel B for use with the 007sp standard (17), 007sp standard, and two in-house control sera from PHE. The IgG concentrations in calibration serum panel B ranged between 0.19 and 6.93, 0.09 and 1.65, 0.16 and 6.68, 0.37 and 63.31, 0.34 and 4.93, 0.21 and 9.81, 0.31 and 30.79, 0.27 and 5.50, 1.49 and 109.5, 0.23 and 14.14, 0.36 and 41.86, 0.44 and 10.73, and 0.15 to 24.66 μg/ml for serotypes 1, 3, 4, 5, 6A, 6B, 7F, 9V, 14, 18C, 19A, 19F, and 23F, respectively. The samples were relabeled before distribution by PHE, and the assigned antibody concentrations of the samples were handled by all laboratories in a blind manner. All laboratories quantified the sera against the presently available 007sp standard. The sera were analyzed for IgG against the different PPSs in two or three independent runs, depending on the laboratory. The number of serotypes assayed ranged from 7 to 23 depending on the laboratory. Either of two assay platforms, MIA or the WHO EIA, was used (for details, see Table 4 and Table S1 in the supplemental material). The assessment of agreement between laboratories and assay platforms was restricted to results measured using 007sp as the standard and to laboratories that reported results for at least 10 serotypes assayed in at least two independent runs.

**TABLE 4:** Participating laboratories and assay platforms^*a*^

Institution	Assay platform(s)	Reference(s)
Institute of Child Health (UCL), London, United Kingdom	WHO EIA	31
National Institute for Health and Welfare (THL), Helsinki, Finland	MIA	20
National Institute of Public Health and the Environment (RIVM), Bilthoven, The Netherlands	MIA	18, 28
Norwegian Institute of Public Health (NIPH), Oslo, Norway	MIA	20
Public Health Agency of Sweden, Stockholm, Sweden	MIA	20
Public Health England (PHE), Manchester, United Kingdom	MIA	18
Quest Diagnostics, Infectious Disease, San Juan Capistrano, CA, USA	MIA	20
Reinier HAGA MDC, Delft, The Netherlands	WHO EIA, MIA	18, 31, 32
St Antonius Hospital, Nieuwegein, The Netherlands	MIA	18, 31, 32
Statens Serum Institut (SSI), Copenhagen, Denmark	MIA	18
University of Alabama, Birmingham, AL, USA	WHO EIA	31

a The laboratories are listed alphabetically, and this order is not associated with designations I to XII.

### Multiplex immunoassay.

The protocols used by each laboratory and the harmonized version are summarized in Table S1.

### Enzyme immunoassay.

The WHO EIAs were essentially performed as described previously by Wernette et al. (31).

### PPS source comparison.

The serotype-specific anti-PPS IgG antibodies were measured in human serum samples utilizing PPS from two different manufacturers, namely, American Type Culture Collection, Manassas, VA, USA (ATCC), and SSI Diagnostica, Hilleroed, Denmark (see Text S1). PPSs were conjugated to beads by the use of the two-step carbodiimide reaction as described previously by Lal et al. (18). MIA was performed according to the protocol of laboratory I (see Table S1), and all samples were analyzed in duplicate. The human serum samples included 97 routine diagnostic serum samples from SSI (unknown donors, unknown vaccination status) and 24 serum samples obtained from WHO; 12 samples from pneumococcal ELISA calibration serum panel A (for use with 89SF), and 12 samples from panel B (for use with 007sp). The panel A samples have assigned values for IgG antibodies against seven serotypes included in this study (4, 6B, 9V, 14, 18C, 19F, and 23F), whereas the panel B samples have assigned values for IgG antibodies against 12 serotypes evaluated in this study (1, 3, 4, 5, 6A, 6B, 7F, 9V, 14, 18C, 19A, 19F, and 23F) (17). IgG concentration data (given as micrograms per milliliter) were log transformed for statistical analysis.

### Conjugation method comparison.

The following two basic conjugation chemistries were employed to conjugate PPSs to carboxylated beads for MIA: the two-step carbodiimide method involving poly-l-lysine (PLL) (18, 19) and the method employing 4-(4,6-dimethoxy-1,3,5-triazin-2-yl)-4-methyl-morpholinium chloride (DMTMM) (20). The conjugation chemistry used by each participating laboratory is described in Table S1.

### Statistical analysis.

All IgG antibody concentrations were expressed as micrograms per milliliter. Each laboratory-measured concentration was calculated as the geometric mean of the concentrations determined in two or three independent runs of each sample in each laboratory. All antibody concentrations were log transformed prior to analysis. Accuracy is defined as the closeness of a laboratory-measured value to the assigned value and is measured using Lin’s coefficient of accuracy (*C_a_*). Precision is a measure of how far a set of observations deviates from a straight line and is quantified using Pearson’s correlation coefficient (*r*). Lin’s concordance correlation coefficient (*r_c_*), which represents a combination of *C_a_* and *r*, was employed to form a single statistic describing both accuracy and precision. Bias is a measure of the directional error of the laboratory-measured value with respect to the assigned value. Within-laboratory variability was assessed by percent coefficient of variation (CV%) of results from the two or three independent measurements. The criterion for acceptable variability was set to a CV value of ≤20%. The parameters chosen and criteria for good agreement between bead-based assays (*r_c_* = ≥0.80) were based on work by Whaley et al. (22). In the analyses of PPS sources and conjugation methods, the antibody results were compared by the use of Deming regression. The results of the regression analyses were divided into categories according to the following criteria: excellent agreement, 1.2 > slope > 0.8; good agreement, 1.4 > slope > 0.6; poor agreement, 1.4 < slope < 0.6.
