# Supplementary figures and images for: Multilaboratory Comparison of Pneumococcal Multiplex Immunoassays Used in Immunosurveillance of Streptococcus pneumoniae across Europe
Source: mSphere. 2019 Nov 27;4(6):e00455-19. doi: 10.1128/mSphere.00455-19 (PMC6881716; doi:10.1128/mSphere.00455-19)

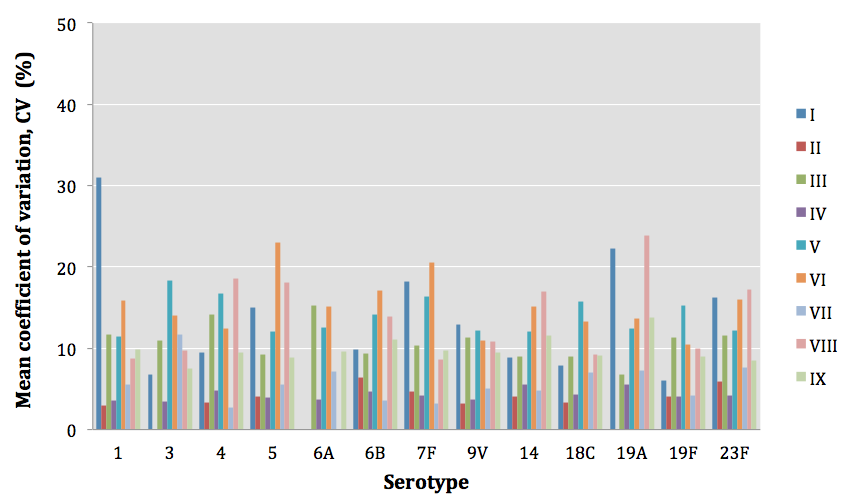

Supplement: FIG S1 [file mSphere.00455-19-sf001.tif]

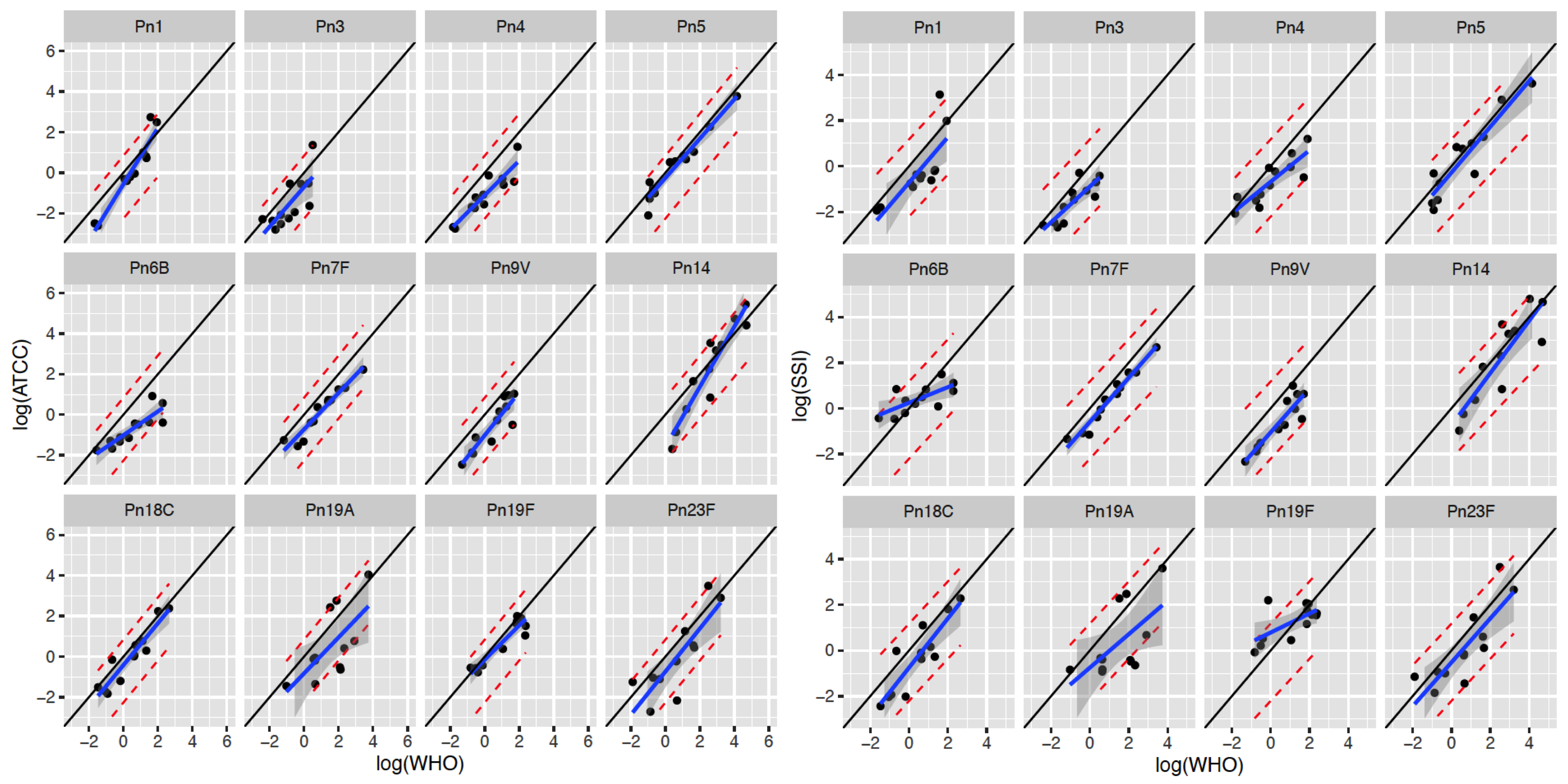

Supplement: FIG S2 [file mSphere.00455-19-sf002.tif]

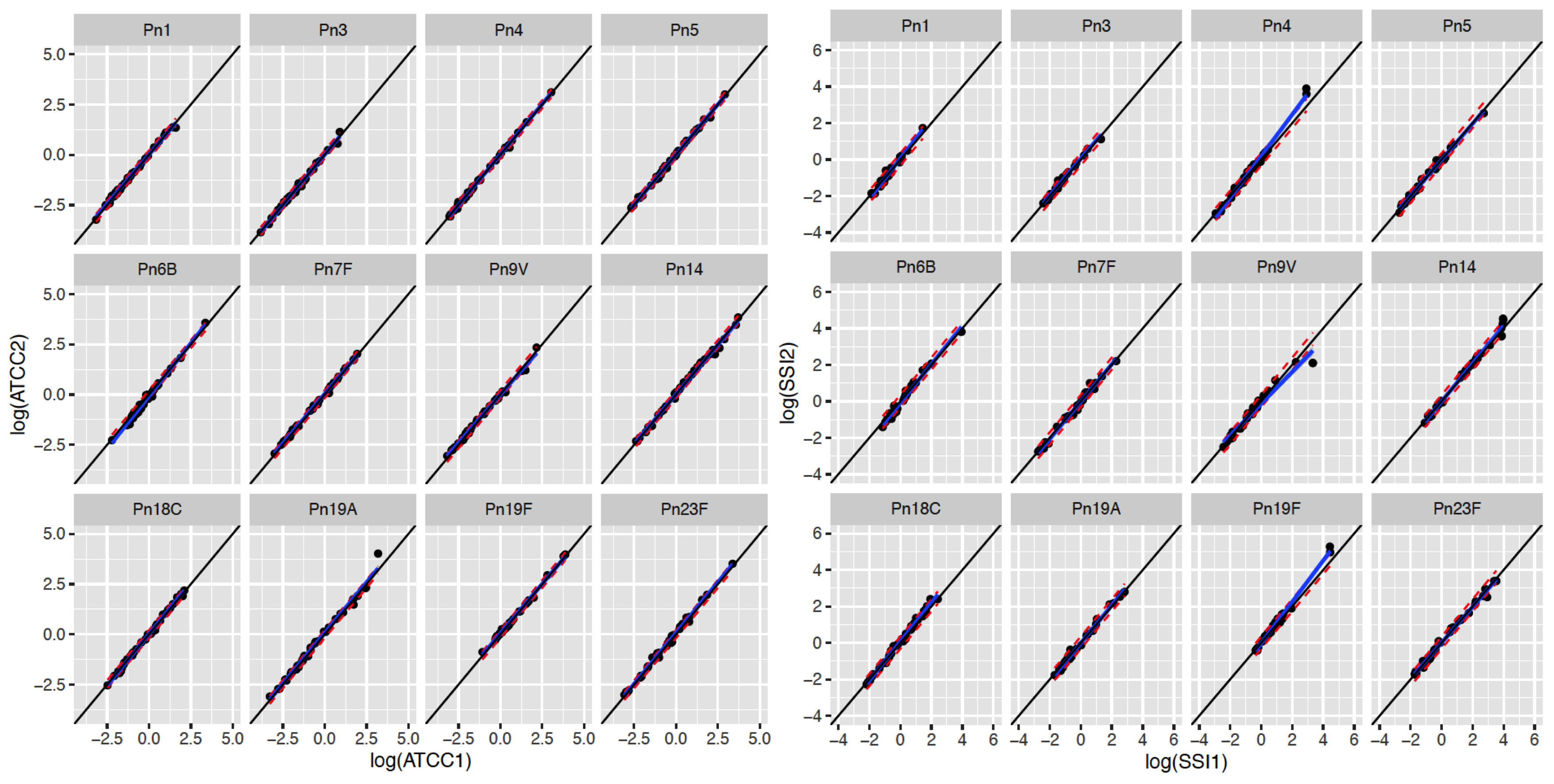

Supplement: FIG S3 [file mSphere.00455-19-sf003.tif]

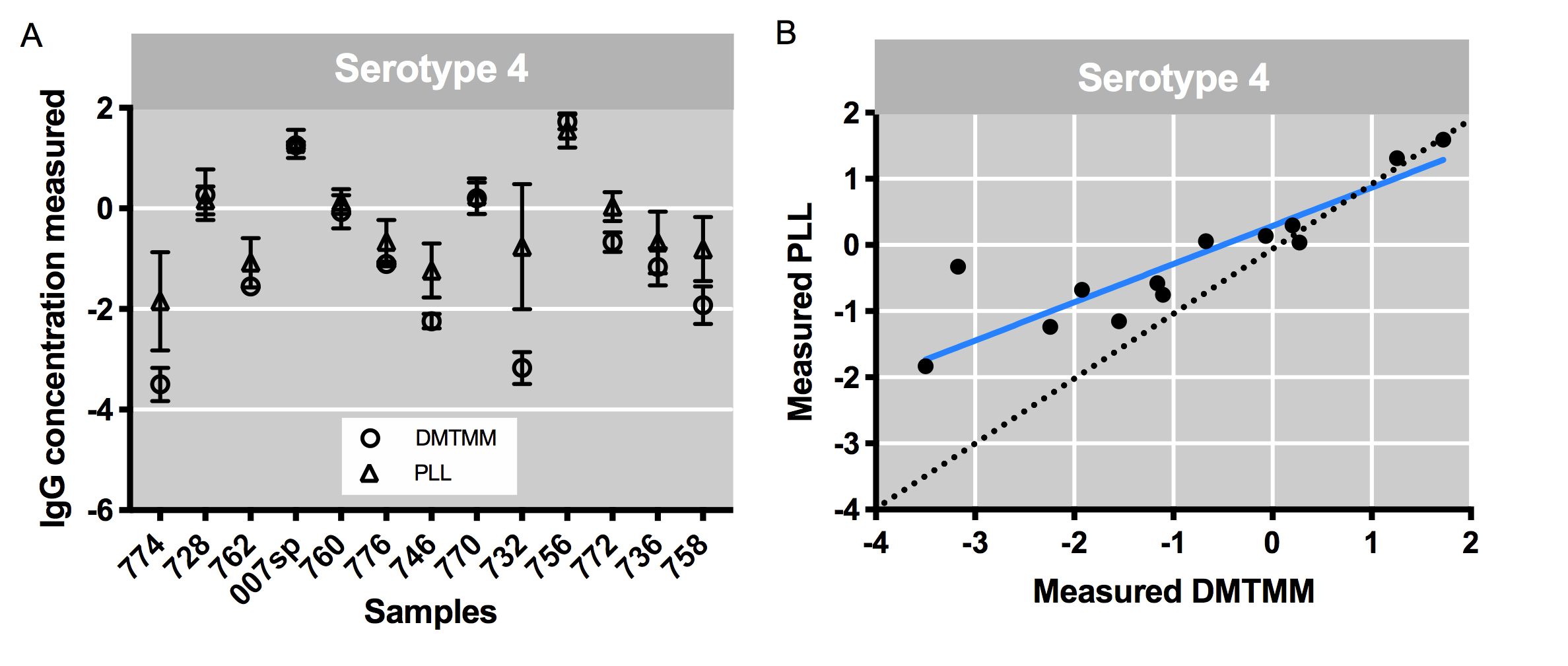

Supplement: FIG S4 [file mSphere.00455-19-sf004.tif]
