# Supplementary material for: Multilaboratory Comparison of Pneumococcal Multiplex Immunoassays Used in Immunosurveillance of Streptococcus pneumoniae across Europe
Source: mSphere. 2019 Nov 27;4(6):e00455-19. doi: 10.1128/mSphere.00455-19 (PMC6881716; doi:10.1128/mSphere.00455-19)
Supplement: TABLE S1 [file mSphere.00455-19-st001.docx]

**Table S1.** Overview of MIA protocols used by participating laboratories

| Protocol at a glance | Harmonized protocol | I | II | III | IV, V, VI | VII | VIII | IX |
| --- | --- | --- | --- | --- | --- | --- | --- | --- |
| Platform | **Bioplex 200, MagPix** | Bioplex | MagPix | Luminex L100/200 | BioPlex, MagPix | Bioplex 200 | Bioplex 200 | MagPix |
| Analysis software | **BioPlex Manager 6.1 /6.0 /4.1.1 or xPONENT 4.2** | BioPlex Manager 6.0 | xPONENT 4.2 | xPONENT 3,1 | BioPlex Manager 4.1 or 6,1 | Bioplex Manager 6.1 | BioPlex Manager 4.1.1 | BioPlex Manager 6.1 |
| Curvefit | **5PL** | 5PL | 5PL | 5PL | 5PL | 5PL | 5PL | 5PL |
| Method of conjugation | **PLL / EDC / Sulpho-NHS or DMTMM** | PLL / EDC / Sulpho-NHS | DMTMM | DMTMM | PLL / EDC / Sulpho-NHS | DMTMM | PLL / EDC / Sulpho-NHS | DMTMM |
| Microspheres | **Microplex© or Magplex©** | Microplex© | Magplex© | Magplex© | Microplex© or Magplex© | Magplex© | Microplex© | Magplex© |
| Blocking agent 1 | **ADHS or NBBS** | NBBS | ADHS | StabilGuard Choice | ADHS | ADHS | NBBS | ADHS |
| Vendor | **Valley Biomedical/Fischer or Biosera** | Fischer | Valley Biomedical | Surmodics | Valley Biomedical | Valley Biomedical | Biosera | Valley Biomedical |
| Conjugate | **Anti-human IgG-RPE (Goat)** | Anti-human IgG-RPE (Goat) | Anti-human IgG-RPE (Goat) | Anti-human IgG-RPE (Goat) | Anti-human IgG-RPE (Goat) | Anti-human IgG-RPE (Goat) | Anti-human IgG-RPE (Goat) | Anti-human IgG-RPE (Goat) |
| Vendor | **Jackson Immunoresearch or Stratech** | Jackson Immunoresearch | Jackson Immunoresearch | Jackson Immunoresearch | Jackson Immunoresearch | Jackson Immunoresearch | Stratech | Jackson Immunoresearch |
| Blocking agent 2 | **CWPS + PPS 22F or CWPS multi** | CWPS + PPS 22F | CWPS + PPS 22F | CWPS multi | CWPS multi | CWPS multi | CWPS + PPS 22F | CWPS multi + PPS 22F |
| Vendor | **SSI (CWPS / CWPS multi) and ATCC (22F)** | SSI and ATCC (22F) | SSI and ATCC (22F) | SSI | SSI | SSI | SSI and ATCC (22F) | SSI and ATCC (22F) |
| Polysaccharides (PPS) | **1,3,4,5,6A,6B,7F,9V,14,18C,19A,19F,23F** | All, except 6A | All, except 3, 6A, 19A | All | All | All | All, except 6A | All |
| Vendor | **ATCC and SSI (7F and 19F)** | ATCC and SSI (7F and 19F) | ATCC | ATCC and SSI (19F) | ATCC and SSI (19F) | ATCC and SSI (6A, 19F) | ATCC and SSI (7F and 19F) | ATCC and SSI (7F and 19F) |
| Beads per PPS / sample | **1750- 5000** | 5000 | 1750 | 800 | 4000 | 1750 | 5000 | 2500 |
| Serum incubation time with blocking agent 2 | **O/N +4°C or 60 min RT** | not submitted | O/N, +4°C | 60 min, RT | 60 min, RT | O/N, +4°C | Up to 5 hours, RT | O/N, +4°C |
| Standard incubation time with blocking agent 2 | **30 min RT / as sample** | not submitted | O/N, +4°C | 60 min, RT | 60 min, RT | 30 min, RT | 20 min, dark&shaking, RT | 30 min, RT |
| Incubation time beads and serum | **30-60 min, dark&shaking, RT** | 30 min, dark&shaking, RT | 60 min, dark&shaking, RT | 20 min, shaking, RT | 30 min, dark&shaking, RT | 60 min, dark&shaking, RT | 20 min, dark&shaking, RT | 60 min, dark&shaking, RT |
| Incubation time conjugate | **20-60 min, dark&shaking, RT** | 30 min, dark&shaking, RT | 30 min, dark&shaking, RT | 20 min, shaking, RT | 20 min, dark&shaking, RT | 60 min, dark&shaking, RT | 20 min, dark&shaking | 60 min, dark&shaking, RT |
