# Supplementary material for: Multilaboratory Comparison of Pneumococcal Multiplex Immunoassays Used in Immunosurveillance of Streptococcus pneumoniae across Europe
Source: mSphere. 2019 Nov 27;4(6):e00455-19. doi: 10.1128/mSphere.00455-19 (PMC6881716; doi:10.1128/mSphere.00455-19)
Supplement: TABLE S2 [file mSphere.00455-19-st002.docx]

| PPS | LAB | I | II | III | IV | V | VI | VII | VIII | IX |
| --- | --- | --- | --- | --- | --- | --- | --- | --- | --- | --- |
| 1 |  | 1.5 | 1.4 | 1.4 | 1.2 | 1.2 | 0.96 | 1.6 | 1.3 | 1.4 |
|  |  | 0.97-2.1 | 1.2-1.7 | 1.2-1.6 | 1.0-1.4 | 0.99-1.5 | 0.75-1.2 | 1.2-1.9 | 0.92-1.7 | 1.2-1.7 |
| 3 |  | 1.1 | NA | 1.2 | 0.76 | 0.78 | 0.44 | 1.1 | 0.99 | 1.2 |
|  |  | 0.49-1.8 |  | 0.63-1.8 | 0.34-1.2 | 0.34-1.2 | 0.044-0.84 | 0.47-1.7 | 0.26-1.7 | 0.56-1.8 |
| 4 |  | 1.0 | 1.2 | 1.4 | 0.80 | 0.50 | 0.37 | 1.3 | 0.81 | 1.2 |
|  |  | 0.71-1.4 | 0.79-1.5 | 1.1-1.6 | 0.52-1.1 | 0.29-0.70 | 0.078-0.65 | 0.97-1.6 | 0.42-1.2 | 0.94-1.4 |
| 5 |  | 1.2 | 1.4 | 1.2 | 1.2 | 1.3 | 1.0 | 1.2 | 1.1 | 1.2 |
|  |  | 0.96-1.4 | 0.97-1.8 | 0.91-1.4 | 0.95-1.4 | 1.1-1.5 | 0.73-1.3 | 0.93-1.5 | 0.88-1.4 | 1.0-1.5 |
| 6A |  | NA | NA | 1.1 | 0.89 | 1.0 | 0.95 | 0.98 | NA | 1.0 |
|  |  |  |  | 0.44-1.7 | 0.33-1.5 | 0.38-1.7 | 0.34-1.6 | 0.33-1.6 |  | 0.44-1.6 |
| 6B |  | 1.1 | 1.4 | 1.4 | 1.3 | 1.3 | 1.2 | 1.5 | 1.5 | 1.5 |
|  |  | 0.72-1.5 | 1.0-1.8 | 1.1-1.7 | 0.96-1.6 | 1.0-1.7 | 0.86-1.6 | 1.1-1.9 | 1.1-1.8 | 1.1-1.8 |
| 7F |  | 0.92 | 1.1 | 1.1 | 0.93 | 1.0 | 0.90 | 1.1 | 1.0 | 1.0 |
|  |  | 0.70-1.1 | 0.76-1.4 | 0.87-1.3 | 0.76-1.1 | 0.88-1.2 | 0.70-1.1 | 0.95-1.2 | 0.86-1.2 | 0.85-1.2 |
| 9V |  | 1.1 | 1.2 | 1.3 | 1.1 | 1.3 | 1.1 | 1.3 | 1.4 | 1.3 |
|  |  | 0.70-1.6 | 0.85-1.6 | 1.0-1.6 | 0.73-1.5 | 0.93-1.6 | 0.72-1.4 | 0.87-1.8 | 0.95-1.8 | 0.91-1.7 |
| 14 |  | 1.3 | 1.5 | 1.3 | 1.3 | 1.4 | 1.1 | 1.5 | 1.3 | 1.4 |
|  |  | 1.0-1.5 | 1.3-1.8 | 1.0-1.5 | 1.1-1.5 | 1.2-1.6 | 0.83-1.4 | 1.3-1.8 | 1.1-1.4 | 1.0-1.7 |
| 18C |  | 1.0 | 1.2 | 1.2 | 1.1 | 1.1 | 0.98 | 1.2 | 1.2 | 1.2 |
|  |  | 0.73-1.3 | 0.90-1.5 | 0.96-1.4 | 0.80-1.4 | 0.84-1.4 | 0.68-1.3 | 0.92-1.5 | 0.88-1.4 | 0.92-1.5 |
| 19A |  | 0.96 | NA | 1.2 | 1.1 | 1.3 | 1.0 | 1.4 | 1.3 | 1.2 |
|  |  | 0.16-1.8 |  | 0.50-1.8 | 0.58-1.6 | 0.84-1.8 | 0.59-1.5 | 0.77-2.1 | 0.70-1.9 | 0.62-1.7 |
| 19F |  | 0.91 | 1.4 | 1.3 | 1.2 | 1.3 | 1.2 | 1.5 | 1.4 | 1.3 |
|  |  | 0.66-1.2 | 1.2-1.6 | 1.2-1.4 | 1.0-1.4 | 1.1-1.4 | 0.99-1.3 | 1.3-1.7 | 1.2-1.7 | 1.1-1.5 |
| 23F |  | 1.1 | 1.2 | 1.3 | 0.94 | 1.1 | 0.97 | 1.3 | 1.2 | 1.3 |
|  |  | 0.64-1.5 | 0.74-1.7 | 0.94-1.6 | 0.54-1.5 | 0.94-1.6 | 0.65-1.3 | 0.80-1.8 | 0.77-1.5 | 0.87-1.7 |

NA = not available.
