# Supplementary material for: Multilaboratory Comparison of Pneumococcal Multiplex Immunoassays Used in Immunosurveillance of Streptococcus pneumoniae across Europe
Source: mSphere. 2019 Nov 27;4(6):e00455-19. doi: 10.1128/mSphere.00455-19 (PMC6881716; doi:10.1128/mSphere.00455-19)
Supplement: TEXT S1 [file mSphere.00455-19-s0001.docx]

**Text S1: supplemental method**

Batch numbers of ATCC and SSI Diagnostica polysaccharides used for comparative analysis

PPS ATCC SSI Diagnostica

1 2101609 QCPS1-4

3 2083298 QCPS3-4

4 2087824 QCPS4-3

5 2090508 CPS5M-7

6B 2095424 CPS6BM-7

7F 41318601 CPS7FM-9

9V 2069508 SCPS9V-1

14 2098654 QCPS14-3

18C P127951-140 TCPSS18C-1

19A 2099856 CPS19AM-6

19F P127908 QCPS19F-2

23F 2099958 CPS23FM-8
